# The *Drosophila* Hox gene *Ultrabithorax* acts in both muscles and motoneurons to orchestrate formation of specific neuromuscular connections

**DOI:** 10.1242/dev.143875

**Published:** 2017-01-01

**Authors:** Christian Hessinger, Gerhard M. Technau, Ana Rogulja-Ortmann

**Affiliations:** Institute of Genetics, University of Mainz, Mainz 55128, Germany

**Keywords:** Motoneurons, Muscle innervation, Segmental patterning, Hox genes, Wnt signalling pathway, *Drosophila*

## Abstract

Hox genes are known to specify motoneuron pools in the developing vertebrate spinal cord and to control motoneuronal targeting in several species. However, the mechanisms controlling axial diversification of muscle innervation patterns are still largely unknown. We present data showing that the *Drosophila* Hox gene *Ultrabithorax* (*Ubx*) acts in the late embryo to establish target specificity of ventrally projecting RP motoneurons. In abdominal segments A2 to A7, RP motoneurons innervate the ventrolateral muscles VL1-4, with VL1 and VL2 being innervated in a *Wnt4*-dependent manner. In *Ubx* mutants, these motoneurons fail to make correct contacts with muscle VL1, a phenotype partially resembling that of the *Wnt4* mutant. We show that *Ubx* regulates expression of *Wnt4* in muscle VL2 and that it interacts with the Wnt4 response pathway in the respective motoneurons. *Ubx* thus orchestrates the interaction between two cell types, muscles and motoneurons, to regulate establishment of the ventrolateral neuromuscular network.

## Abstract (precis)

**Summary:** Ultrabithorax controls correct innervation of ventrolateral muscles by coordinating Wnt4 ligand expression in muscles with the signalling pathway response in motoneurons.

## INTRODUCTION

Establishment of region-specific muscle innervation patterns in animals is crucial for fulfilling functions specific to various body segments along the anteroposterior axis, such as locomotion or mating. How motoneuronal networks are regionally adjusted is a long-standing question in developmental neurobiology. The highly conserved Hox transcription factors are known to govern decisive aspects of motoneuronal network formation in flies and vertebrates (Philippidou and Dasen, 2013). In mice, they regulate specification of motoneuron (MN) pools and control their projection patterns (Bell et al., 1999; Catela et al., 2016; Dasen et al., 2005; Lacombe et al., 2013; Philippidou et al., 2012; Studer et al., 1996). In *Drosophila*, they regulate, among other processes, survival and identity of leg MNs (Baek and Mann, 2009; Baek et al., 2013). Whereas many studies have investigated the role of Hox-mediated regulatory networks during specification and differentiation of neurons, the molecular mechanisms underlying these actions are just beginning to be discovered. Recent studies in flies provided the first hints of a combined regulation of Hox-controlled targets in MNs and muscles. For example, the Hox gene *Deformed* (*Dfd*) is required in both establishment of the larval neuromuscular feeding unit and later in regulation of specific motoneuronal effector genes such as that encoding Ankyrin2-XL (Friedrich et al., 2016). Another study on larval crawling showed that this behaviour requires characteristic peristaltic movements of abdominal muscles, whereas movement of thoracic and head segments follows a rather different pattern (Dixit et al., 2008). These distinct patterns of movement rely on defined muscle architecture and precise motoneuronal innervation thereof. Regionally distinct muscle patterns are established early in development under the influence of Hox genes (Michelson, 1994) and ubiquitous expression of *Ultrabithorax* (*Ubx*), which specifies the first abdominal segment, results in thoracic segments exhibiting an abdominal peristaltic pattern (Dixit et al., 2008). Interestingly, providing *Ubx* either in neurons or in muscles alone does not produce the same effect. Although Dixit et al. showed a requirement for *Ubx* in both tissues, it remains unclear how this Hox gene establishes a functional neuromuscular system in the abdomen (Dixit et al., 2008). In particular, how expression of various factors involved in motoneuronal targeting of specific muscles is coordinated between these two tissues is still largely unknown.

In order to find and connect to their target muscles, MNs, upon being individually specified, need to extend their axons in a spatially and temporally highly regulated manner and navigate through a complex environment of different signals (Prokop, 1999). Several families of guidance molecules have been identified in various model organisms (Dickson, 2002; Nose, 2012). More recently, involvement of factors classically characterized as morphogens belonging to the Wnt, Hedgehog and TGFβ superfamilies have been shown to provide positional information and interact with pathfinding processes in different species (Charron et al., 2003; Inaki et al., 2007; Klassen and Shen, 2007; Lyuksyutova et al., 2003; Parker et al., 2006; Serpe and O'Connor, 2006). These factors provide signals to growth cones that guide them to the correct target or help in the decision of where to make synapses (Marqués, 2005; Schnorrer and Dickson, 2004). Although the components, either expressed at the cell surface or secreted, have been characterised to a large extent (Kurusu et al., 2008; Nose, 2012), the underlying transcriptional programmes required to orchestrate the expression of these guidance factors and the corresponding neuronal responses remain less well understood (Santiago and Bashaw, 2014; Zarin et al., 2014).

Here, we present data showing that the *Drosophila* Hox gene *Ubx* orchestrates the formation of a specific neuromuscular connection in abdominal segments of the *Drosophila* embryo. It does so by regulating *Wnt4* expression in the muscle and by interacting with the Wnt4 signalling pathway in the corresponding MNs. Our data demonstrate that, through its dual function, *Ubx* coordinates communication between muscles and motoneurons to establish correct neuromuscular connections.

## RESULTS

### Segment-specific differences in innervation of ventrolateral muscles

**Fig. 1.**
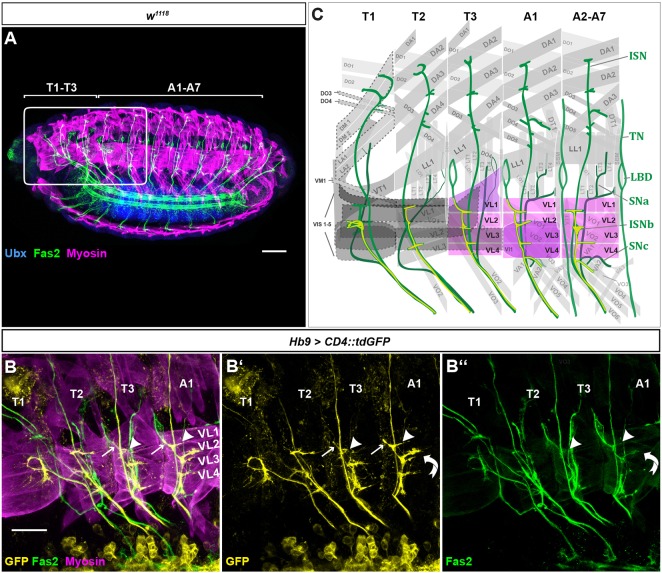
**Morphological characterisation of VL muscles and their innervation pattern in the late embryo.** Shown are thoracic (T1-T3) and abdominal (A1-A7) segments with focus on the innervation of the VL muscle group. (A) External view of a wild-type whole-mount embryo (*w^1118^*). Stained are motoneuronal tracts using anti-Fas2 (green), muscles using anti-Myosin (magenta), and anti-Ubx (blue). Ubx is expressed at high levels within the CNS. The white rectangle marks the area depicted in the scheme in C. (B-B″) Filet preparation of an early stage 17 embryo. The innervation pattern is visualised using membrane-bound UAS-*CD4::tdGFP* expressed under *Hb9-*Gal4 control. Arrowheads mark the innervations of VL1 in T3 and A1. Noteworthy are the segment-specific differences with regard to the ISNb contact with LBD in T3 and A1 (arrows in B and B′). The curved arrow indicates innervation of muscle VI1 by the DC MN in A1. (C) A scheme of the internal view on the muscle field. Relevant muscles are coloured in magenta. Muscles with segment-specific modifications in T3 and A1 are coloured in purple. Those muscles for which morphology and identity were difficult to determine are encircled by a dashed line. The transverse nerve (TN) is missing in thoracic segments and A1. Anterior is to the left in all panels, dorsal is up. SN, segmental nerve. Scale bars: 40 µm (A); 20 µm (B).

The ventrolateral (VL) muscles of embryonic abdominal segments A2 to A7 as well as their innervation pattern are well-described (Bate, 1993; Choi et al., 2004; Hoang and Chiba, 2001; Hooper, 1986; Landgraf et al., 1997; Mauss et al., 2009; Michelson, 1994). To explore whether this group of muscles provides a good model for investigating mechanisms leading to regional differences in motoneuronal innervation, we analysed the VL neuromuscular system in the currently less well-characterised thoracic segments T2 and T3, and in A1 of early stage 17 embryos. Muscles VL1, 2, 3 and 4 show only minor morphological changes between segments along the anteroposterior axis (Fig. 1A). They extend parallel to one another and are morphologically similar. In segments A1 to A7, they insert at adjacent muscle insertion sites. In T3, we find VL1 to extend dorsally inserting together with the A1 lateral longitudinal muscle 1 (LL1) into the T3/A1 apodeme (Fig. 1C). Segment T2 exhibits only three VL muscles, VL1-3 (Bate, 1993). These muscles lie somewhat diagonal compared with their counterparts in more posterior segments.

VL muscles are innervated, among other MNs, by RP1, 3, 4 and 5, which derive from the neuroblast NB3-1 (Bossing et al., 1996; Landgraf et al., 1997). We used the *Hb9*-Gal4 line, which is active in all postmitotic RP MNs and a limited set of interneurons and dorsal MNs (Broihier et al., 2002), in combination with *CD4::tdGFP* to visualize the axonal projections of *Hb9* (*exex*)-positive MNs. These MNs cross the posterior segment border and project into the intersegmental nerve branch b (ISNb) of the next segment, so that the MNs of segment A1 innervate VL muscles in segment A2 (Broihier et al., 2002; Landgraf et al., 1997; Matthes et al., 1995). In segments A2 to A7, muscle VL1 receives a stereotypical ‘T’-shaped contact by RP5. It is also innervated by the so-called V neuron, and possibly a further MN (Choi et al., 2004; Hoang and Chiba, 2001; Inaki et al., 2007, 2010; Landgraf et al., 1997; Mauss et al., 2009). From here on, we refer to this group of VL1-innervating MNs as VL1-MNs. In segments T3 and A1, one or more of the VL1-MNs extend an additional dorsal projection that contacts the lateral bipolar dendritic neuron (LBD) (Fig. 1B,C). By contrast, in A2-A7 the VL1-MNs do not make this contact and the LBD fuses with the transverse nerve (TN), which is not present in thoracic segments and A1 (Gorczyca et al., 1994). In T3 to A7, RP1 and RP4 innervate the ventral oblique muscles 2 (VO2) and 1 (VO1), respectively (Choi et al., 2004; Mauss et al., 2009). VL3 and VL4 are innervated by RP3, which forms a fine contact in the cleft between these two muscles (Landgraf et al., 1997; Mitchell et al., 1996). This contact is also similar in T2 to A7. In T2, the ISNb innervates VL1-3 similarly to innervation in abdominal segments (Fig. 1C). Furthermore, the A1-specific ventral internal muscle 1 (VI1) is innervated by the DC1 MN (Matthes et al., 1995), which projects through the ISNb and is also labelled in *Hb9*>*CD4::tdGFP* embryos (Fig. 1B,C).

Thus, in segments T3 and A1, muscle VL1 and the MNs that innervate it show deviations from the typical abdominal pattern. These segments are characterized by rather high overall expression levels of the Hox gene *Ubx*, especially in the ventral nerve cord (VNC). Because Hox genes have generally been shown to be involved in late events of CNS maturation (Friedrich et al., 2016; Miguel-Aliaga and Thor, 2004; Rogulja-Ortmann et al., 2008), we wondered whether *Ubx* influences axonal projections of and target muscle selection by VL1-MNs.

### A requirement for *Ubx* in establishment of correct motoneuron contacts on VL muscles

**Fig. 2.**
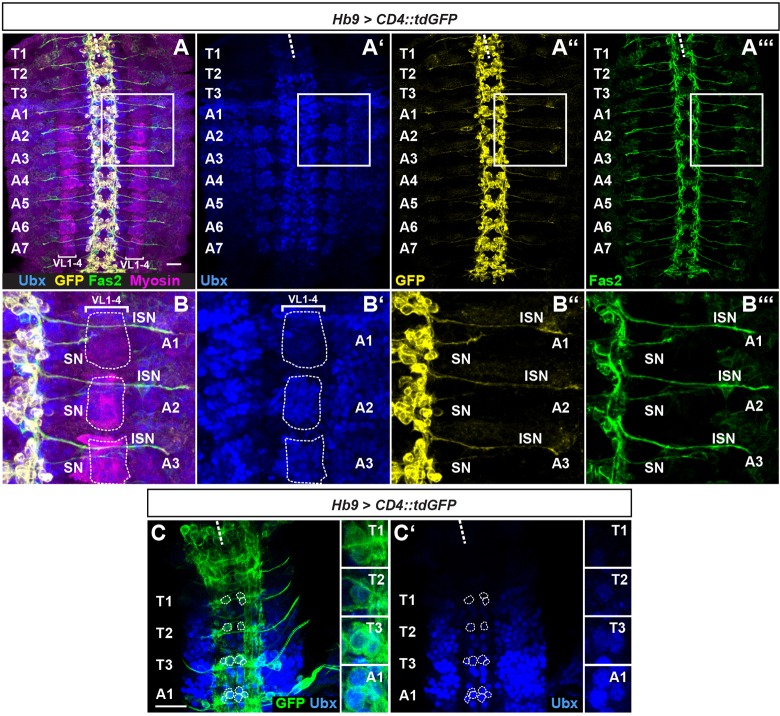
**Ubx expression in VL muscles and RP neurons.** (A-A‴) Shown is a filet preparation of a late stage 14 embryo with the indicated genotype and stained with anti-Ubx (blue), anti-GFP (yellow), anti-Fas2 (green) and anti-Myosin (magenta). Ubx expression can be observed in VL1-4 of A1 to A7. (B-B‴) Magnified view of the boxed area in A. Muscles VL1-4 are encircled with a white dashed line. In A1, expression levels are lower than in A2 (B′). Growth cones of the ISN and SN have entered the peripheral muscle field (B″,B‴). (C) Filet preparation of an early stage 17 embryo with the indicated genotype stained with anti-Ubx (blue) and anti-GFP (green). Ubx is expressed within RP MNs (encircled) until late developmental stages. Highest expression levels are observed in A1, whereas T2 and T3 show weak Ubx expression (see insets). White dashed line marks the midline. Anterior is up in all images. SN, segmental nerve. Scale bars: 20 µm.

We first examined Ubx expression in the VL muscles and RP MNs in more detail, because the RP MNs were the only VL1-MNs that we could address unambiguously by using the *dHb9*-Gal4 line. At late stage 14, when motoneuronal axons enter the muscle field, Ubx is not expressed in the thoracic VL muscles (Fig. 2A). In A1, these muscles show low Ubx levels (Fig. 2B). Strong expression extends from A2 to A4, whereas it gets progressively weaker in the posterior abdominal segments A5 to A7. Interestingly, Ubx expression levels in the VNC showed a shift of one segment to the anterior compared with the muscles: we observed high levels in A1 and these were progressively reduced towards posterior segments (Fig. 2A). Thus, Ubx expression levels in a particular segment of the VNC appear to correlate with the levels in the muscles of the next posterior segment. We show this in more detail for the thorax and anterior abdomen: RP MNs show low Ubx levels in segments T2 and T3, whereas Ubx levels are high in these neurons in A1 (Fig. 2C, compare with Fig. 2B). This Ubx expression pattern showed an intriguing correlation with the RP MN axonal projection patterns described above, where RP axons cross the posterior segment border and innervate VL muscles of the adjacent posterior segment.

**Fig. 3.**
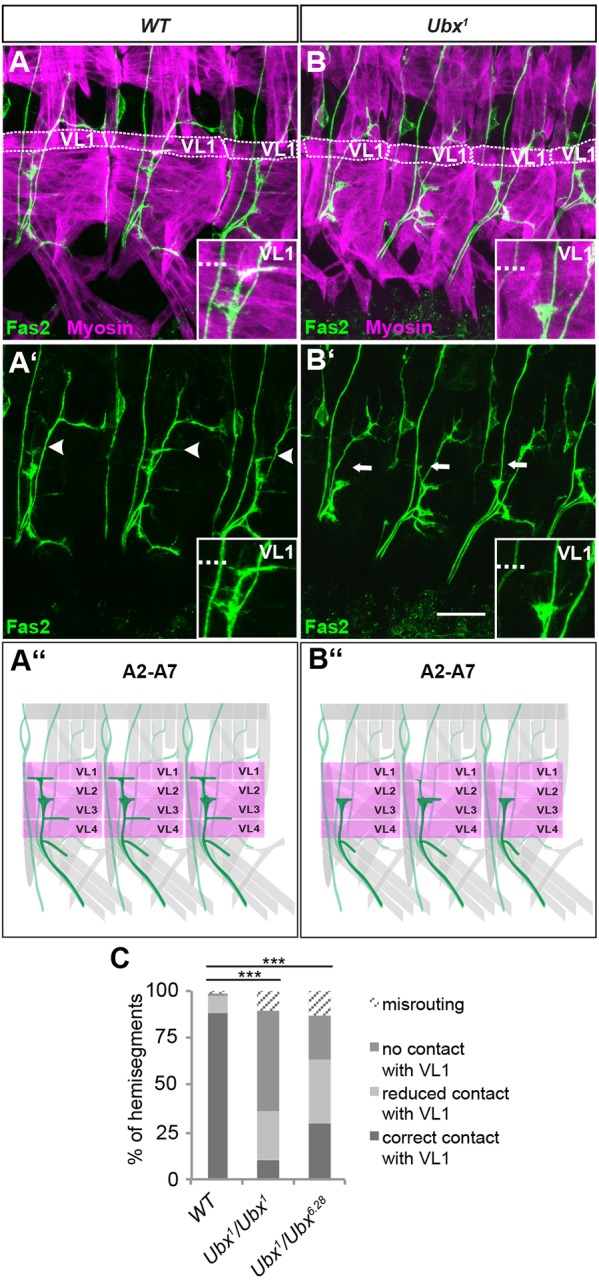
***Ubx* contributes to the correct innervation pattern of the VL muscle group.** (A-B′) Shown are filet preparations of early stage 17 embryos stained with anti-Fas2 (green) and anti-Myosin (magenta). The genotypes are indicated above each panel. (A,A′) In wild-type embryos (WT), VL1-MNs form a T-shaped ending on VL1 in segments A2 to A7 (indicated by white arrowheads). (B,B′) Homozygous *Ubx^1^* mutants show defective innervation of VL1 (white arrows) in A2 to A7. In A and B, VL1 muscles are encircled with a dotted line. In all insets, the dashed line marks the ventral VL1 border. Anterior is to the left, dorsal is up in all images. Scale bar: 20 µm. (A″,B″) Schematics of the observed defects. ISNb is shown in dark green and the VL muscles in magenta. (C) Quantification of the ISNb defects in the different genetic backgrounds. *Ubx* mutants show significant defects compared with WT with regard to VL1 innervation. Data are presented as categories. For statistical analysis, correct contacts were compared with the combination of wrong contacts (misrouting, no contact with VL1, reduced contact with VL1) and significance of the data sets was tested using the χ^2^-test. ***P<0.005. WT: *n*=125; *Ubx^1^*/*Ubx^1^*: *n*=97; *Ubx^1^*/*Ubx^6.28^*: *n*=95 (n, number of evaluated hemisegments).

To test whether *Ubx* plays a role in regionalising VL muscle innervation, we examined *Ubx* null mutants. The terminal differentiation of MN contacts on VL muscles did indeed show segment-specific defects. VL innervation in T3 and A1 did not seem to be affected, possibly due to low Ubx expression levels in VL muscles and the RP MNs of segments T2 and T3 that innervate them (Fig. S1). In abdominal segments A2 to A7, however, we found that innervation of VL1 was either lost or strongly reduced (Fig. 3B). We assigned different categories to characterise the phenotype in more detail. The ‘T’-shaped connection normally seen on VL1 in wild-type embryos was classified as ‘correct contact on VL1’ (Fig. 3A). In *Ubx^1^* mutants, only 10.3% of analysed hemisegments (*n*=97) fall into this category (Fig. 3C; Table S1). The other connections were either reduced (category ‘reduced contact with VL1’; 25.8%; only thin Fas2 signal on VL1 that does not bifurcate in a ‘T’ shape, see insets in Fig. 3B,C) or completely lost (category ‘no contact on VL1’; 53.6%; Fig. 3B,C). The category ‘misrouting’ comprises innervations that could not be attributed to any of the above categories and often involved ectopic contacts with other nerves, most often with the TN. It also included completely aberrant projections, which spread over the VL muscle field. The effects described above in the *Ubx^1^* null mutants were also seen in transheterozygote *Ubx^1^/Ubx^6.28^* animals (Fig. 3C; Table S1), implying a specific and novel role for *Ubx* in this process.

*Ubx* and the abdominal Hox gene *abdominal A* (*abdA*) have been shown to function redundantly in several contexts (Dixit et al., 2008; Michelson, 1994). Although *Ubx* mutants alone show a strong VL1 innervation phenotype, we wanted to test whether *abdA* makes any contribution to this developmental event. As anticipated, VL1 innervation showed no significant changes in *abdA^MX1^* mutants (Fig. S2; Table S1).

To exclude the possibility that the VL1 innervation phenotype observed in *Ubx* mutants is due to loss or temporal mis-specification of RP MNs, we performed anti-Hb9 staining (Broihier et al., 2002) (Fig. S3). All RP MNs were present from A1 to A7 in *Ubx^1^* mutants. Specifically RP5, which co-innervates VL1, could clearly be visualised with anti-Hb9 and anti-Cut double staining (Tran and Doe, 2008). Taken together, these data indicate an as-yet-unknown role of *Ubx* in regulating innervation of VL muscles.

### *Ubx* regulates muscle-specific expression of factors required for proper VL1 innervation

We next investigated by which mechanism *Ubx* might regulate VL muscle innervation. The reduced innervation of VL1, with VL1-MNs stalling on VL2, that we observed in *Ubx* mutants was also reported for embryos mutant for *Wnt4*, a member of the Wnt family of signalling molecules (Inaki et al., 2007) (Fig. S2; Table S1). Furthermore, the same study revealed differential *Wnt4* expression between VL1 and VL2, with higher expression levels in VL2. *Sulf1*, a sulfatase implicated in the regulation of Wnt and BMP gradients at the neuromuscular junction (NMJ) (Dani et al., 2012; Inaki et al., 2007), was also found to be expressed at higher levels in VL2 than in VL1 (Inaki et al., 2007). As the *Ubx* and *Wnt4* mutant phenotypes are remarkably alike, and as Hox genes have been shown to regulate *Wnt4* in the visceral mesoderm of *Drosophila* embryos (Graba et al., 1995), we wondered whether *Ubx* might be regulating *Wnt4* and *Sulf1* expression in the VL muscles.

**Fig. 4.**
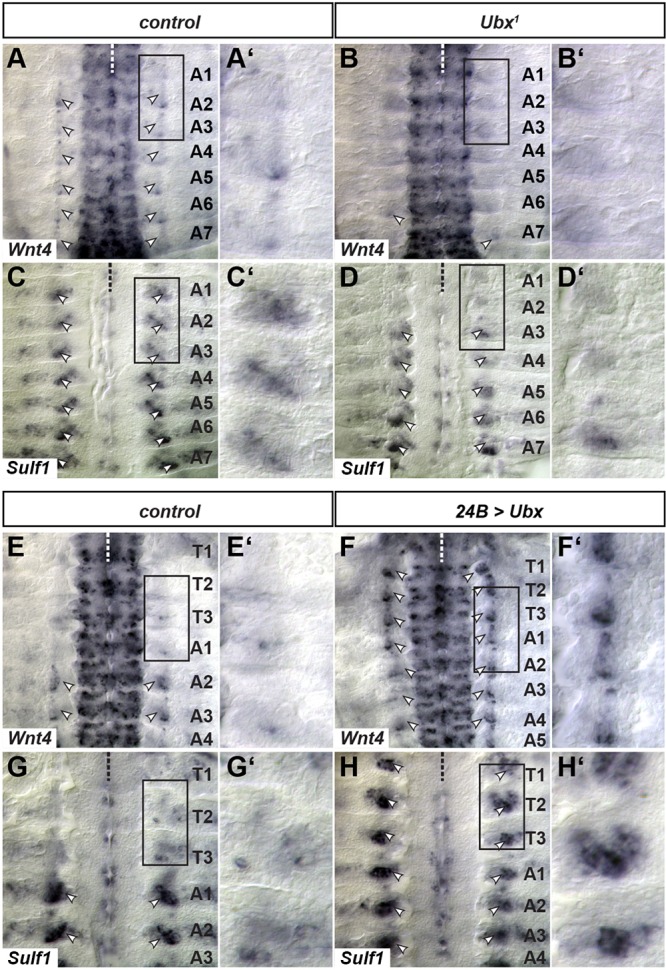
***Ubx* is necessary and sufficient for the expression of *Wnt4* and *Sulf1*.** Shown are filet preparations of late stage 14 embryos after *in situ* hybridisation against *Wnt4* and *Sulf1*. Arrowheads indicate the signal of the respective factor in the VL muscle field. To the right, higher magnifications of areas marked with the black rectangle are shown. (A,A′) The expression of *Wnt4* can be observed as a stripe in VL2 and VA1 (arrowheads) in balanced embryos (controls) within segments A2-A7. In A1, this expression is only weak or not detectable. (B,B′) In *Ubx^1^* mutants, *Wnt4* expression in muscles is almost completely missing, whereas the expression in the CNS is not detectably affected. (C,C′) In control, i.e. balanced, embryos the expression of *Sulf1* can be detected in VL muscles, including VL2 (arrowheads). Furthermore, it can be detected in more dorsal regions, which are probably the lateral-transverse muscles. (D,D′) In *Ubx^1^* mutants, the expression is reduced in a graded fashion, whereby only the expression levels in A6 and A7 appear equal to those in control embryos. In A1 and A2, expression is completely abolished and in A3-A5 it is clearly reduced. (E,E′) Thoracic segments lack expression of *Wnt4* in VL muscles under wild-type conditions. (F,F′) Mesodermal expression of *Ubx* in *24B>Ubx* embryos results in ectopic expression of *Wnt4* in thoracic segments (arrowheads). (G,G′) Thoracic segments show very low levels of *Sulf1* expression under wild-type conditions. (H,H′) Mesodermal expression of *Ubx* in *24B>Ubx* embryos results in ectopic expression in thoracic segments (arrowheads). The dashed line marks the ventral midline. Anterior is up in all images.

In wild-type embryos, *Wnt4* shows a graded expression within the VNC, having the highest levels in the most posterior segments (Fig. 4A). At late stage 14, *Wnt4* shows strongest expression in muscles VL2 and VA1 (ventral acute muscle 1) (Fig. 4A) (Inaki et al., 2007; Nose, 2012) and is subsequently downregulated. From stage 13 onwards, *Sulf1* also shows higher expression levels in VL2 than in VL1, as already reported (Inaki et al., 2007; Nose, 2012) (Fig. 4C). In *Ubx* mutants, *Wnt4* expression in muscles VL2 and VA1 is lost (Fig. 4B). In addition, *Sulf1* is strongly reduced in a graded manner, with a complete loss in segments A1 and A2 (Fig. 4D), showing that expression of both factors requires *Ubx*. We also examined *Wnt4* and *Sulf1* expression when *Ubx* is provided in VL muscles of more anterior segments, where it is normally not expressed. Ectopic *Ubx*, driven by *24B*-Gal4, is sufficient to induce *Wnt4* and *Sulf1* expression in thoracic segments and in A1 (Fig. 4E-H′). Thus, *Ubx* is necessary and sufficient to activate expression of the morphogen *Wnt4* and of a potential Wnt4 modifier, *Sulf1*, in VL muscles, and might thereby control innervation of VL1.

### Wnt4 can activate the canonical Wnt signalling pathway in VL1-innervating motoneurons

**Fig. 5.**
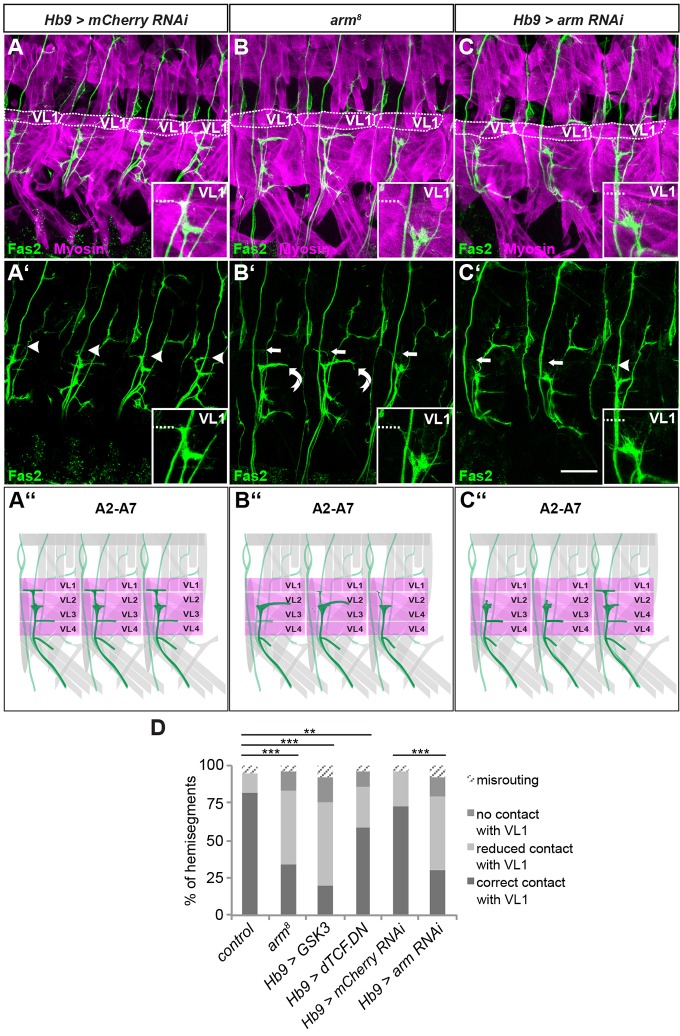
**Manipulating downstream components of canonical Wnt signalling in *Hb9*-positive MNs causes VL1 innervation defects.** (A-C′) Shown are filet preparations of early stage 17 embryos stained with anti-Fas2 (green) and anti-Myosin (magenta). Genotypes are indicated above each panel. (A,A′) In control embryos (*Hb9*>*mCherry* RNAi), VL1-MNs make a T-shaped ending on VL1 (white arrowheads, see insets). (B,B′) In *arm^8^* mutants, innervation of VL1 is strongly reduced (white arrows) and that of VL2 thicker (curved arrow). (C,C′) Knockdown of *arm* in *Hb9*-positive MNs produces similar defects in VL1 innervation (white arrows). In A, B and C, VL1 muscles are encircled with a dotted line. In all insets, the dashed line marks the ventral VL1 border. Scale bar: 20 µm. Anterior is to the left and dorsal is up in all images. (A″-C″) Schematics of the observed defects. The ISNb is coloured in dark green and the VL muscles in magenta. (D) Quantification of the ISNb defects in the different genetic backgrounds. Mutants for *arm^8^* and overexpression of UAS-*arm* RNAi, UAS-*GSK3* or UAS-*dTCF.DN* show significant defects in VL1 innervation compared with control embryos (*Hb9*-Gal4 >UAS-*CD4::tdGFP* or *Hb9*>*mCherry* RNAi). For statistical analysis, correct contacts were compared with the combination of wrong contacts (misrouting, no contact with VL1, reduced contact with VL1) and significance of the data sets was tested using χ^2^-test. **P<0.05, ***P<0.005. *Hb9*-Gal4>UAS-*CD4::tdGFP*: *n*=81 (control); *arm^8^*: *n*=143; *Hb9*-Gal4>UAS-*dGSK3*: *n*=94; *Hb9*-Gal4>UAS-*dTCF.DN*: *n*=105; *Hb9*-Gal4>UAS-*mCherry* RNAi: *n*=78; *Hb9*-Gal4>UAS-*arm* RNAi: *n*=142 (n, number of evaluated hemisegments).

The aforementioned study on control of VL1 innervation showed that Wnt4 requires two Wnt receptors in this context, *Frizzled 2* (*Fz-2*; *fz2 –FlyBase*) and *Derailed 2* (*Drl-2*). Both receptors exhibit mutant phenotypes similar to *Wnt4* (Inaki et al., 2007). However, it remained unclear whether Wnt4 secreted from muscles activates the canonical signalling pathway in the MNs to ensure correct VL1 innervation. To address this question, we examined embryos mutant for the *Drosophila* β-catenin homologue *armadillo* (*arm*), a key transducer of Wnt signalling. We used *arm^8^*, a weak temperature-sensitive allele that lacks *arm* function in epidermal and neuronal Wingless (Wg) signalling but retains it in adherens junctions (Jones et al., 2010; Loureiro and Peifer, 1998; Peifer and Wieschaus, 1990). Because of the high load of maternal *arm* product, these embryos develop until the end of embryogenesis with only mild patterning defects compared with null alleles that eliminate the zygotic gene product (Jones et al., 2010; Loureiro and Peifer, 1998; Peifer and Wieschaus, 1990). The observed effects on epidermal patterning in *arm^8^* mutants can therefore be attributed to very late defects caused by disruption of canonical Wnt signalling. Thus, if the effect on VL muscle innervation is due to late canonical signalling and not to defects in cell adhesion, we would expect to observe a phenotype in *arm^8^* mutants. Indeed, *arm^8^* mutants exhibited defects in VL1 innervation at restrictive temperatures (Fig. 5B). MN contacts with VL1 were often reduced or not present, and correct contacts were found in only 34.3% (*n*=143) of analysed hemisegments (Fig. 5D; Table S1). Instead, the contacts on VL2 were often strongly expanded. These defects were highly specific to VL1-MNs, as we were unable to find obvious phenotypes in the rest of the motoneuronal system or in muscle morphology. Loss of *arm* function in the canonical Wnt signalling pathway thus causes phenotypes similar to those of *Ubx* and *Wnt4* mutants, suggesting that Wnt4 secreted from muscles activates the canonical Wnt signalling pathway in the VL1-MNs. To address more precisely whether activation of Wnt4 signalling is in fact required in these MNs, we performed an *arm* RNAi knockdown using *Hb9*-Gal4. Correct VL1 innervation was significantly reduced to 29.6% (*n*=142) in these embryos (Fig. 5C,D; Table S1), whereas projections of other MNs were not affected. We also analysed the role of further canonical Wnt signalling pathway components in VL1-MNs by overexpressing the negative regulator Glycogen Synthase Kinase-3 (GSK3; Shaggy *–* FlyBase) and a dominant-negative construct (*dTCF.DN*) of the Wnt effector *TCF* (*pan –* FlyBase) (Fig. 5D; Table S1). Indeed, with only 20.2% (*n*=94) and 59% (*n*=105) of correct contacts on VL1, GSK3 and TCF.DN, respectively, both induced significant defects specifically in targeting of VL1 (Fig. 5D; Table S1). Together, these results indicate that canonical Wnt signalling is likely required cell-autonomously in VL1-MNs for correct targeting of this muscle.

### The *Wnt4* signalling pathway and *Ubx* are required in postmitotic neurons for correct VL1 innervation

**Fig. 6.**
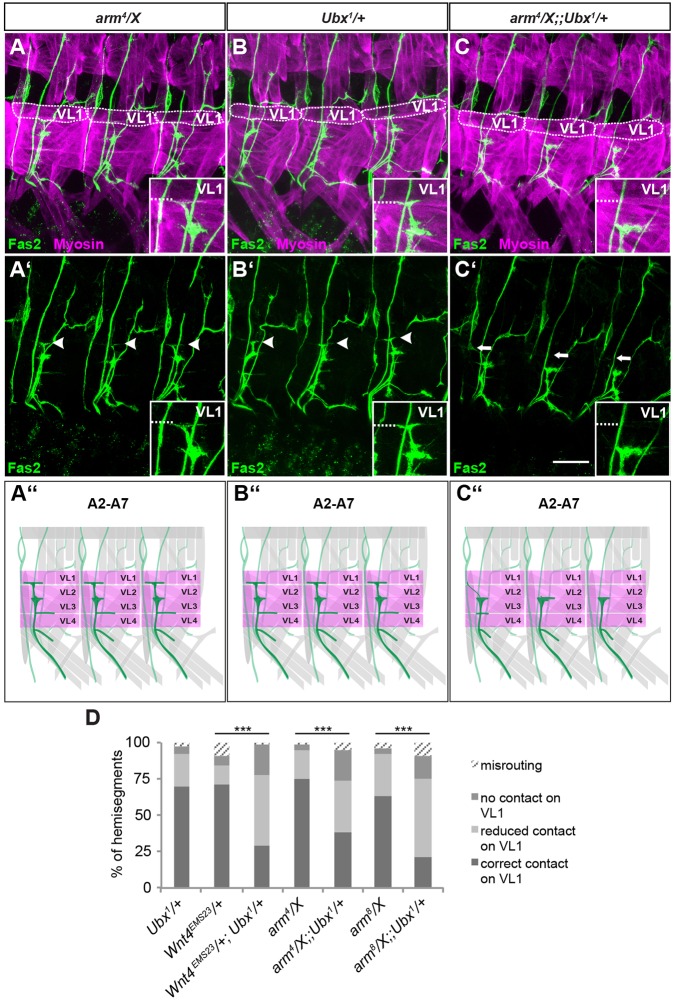
***Ubx* genetically interacts with the Wnt4 pathway to control proper innervation of VL1.** (A-C′) Shown are filet preparations of embryos in early stage 17 stained with anti-Fas2 (green) and anti-Myosin (magenta). The genotypes are given above each panel. (A,A′) In heterozygous *arm^4^/X* controls, VL1 innervation shows a wild-type pattern (white arrowheads, see also inset). (B,B′) In heterozygous *Ubx^1^/+* controls, innervation of VL1 is also unchanged (white arrowheads, see also inset). (C,C′) In *arm^4^*/*X*;;*Ubx^1^*/*+* double-heterozygous embryos, innervation of VL1 is defective in A2-A7 (white arrows, see also inset). In A, B and C, VL1 muscles are encircled with a dotted line. In all insets, the dashed line marks the ventral VL1 border. Anterior is to the left, dorsal is up in all images. Scale bar: 20 µm. (A″-C″) Schematics of the observed defects. ISNb is shown in dark green and the VL muscles in magenta. (D) Quantification of the ISNb defects in the evaluated genetic backgrounds. Genetic interactions between *Ubx* and *Wnt4*, the *arm^4^* null allele and *arm^8^* as a specific mutant of canonical Wg signalling were tested. All double-heterozygous mutants show significant defects compared with their single-heterozygous controls with respect to VL1 innervation. Correct contacts were tested versus the combination of wrong contacts (misrouting, no contact with VL1, reduced contact with VL1) and significance of the data sets was tested using χ^2^**-**test. ***P<0.005. *Ubx^1^*/*+*: *n*=123; *Wnt4^EMS23^*/*+*: *n*=156; *Wnt4^EMS23^*/*+*;*Ubx^1^*/*+*: *n*=103; *arm^4^*/*X*: *n*=94; *arm^4^*/*X*;;*Ubx^1^*/*+*: *n*=107; *arm^8^*/*X*: *n*=60; *arm^8^*/*X*;;*Ubx^1^*/+: *n*=70 (n, number of evaluated hemisegments).

We then tested whether *Ubx* interacts with *Wnt4* and *arm* at the genetic level in this specific developmental context. Heterozygotes of *Ubx^1^*, *Wnt4^EMS23^*, *arm^8^* and the null mutant *arm^4^* showed only a slight decrease in correct innervation of VL1 compared with wild type (Fig. 6A,B,D; Table S1). This changed considerably in *Wnt4^EMS23^*/+; *Ubx^1^*/+, *arm^4^*/X;;*Ubx^1^*/+ or *arm^8^*/X;;*Ubx^1^*/+ double heterozygotes, in which correct contacts on VL1 were significantly decreased to 29.1% (*n*=103), 38.3% (*n*=107) and 21.4% (*n*=70), respectively (Fig. 6C,D; Table S1). Taken together, these data suggest that *Ubx* interacts with the canonical Wnt4 signalling pathway for correct innervation of VL1 by VL1-MNs.

**Fig. 7.**
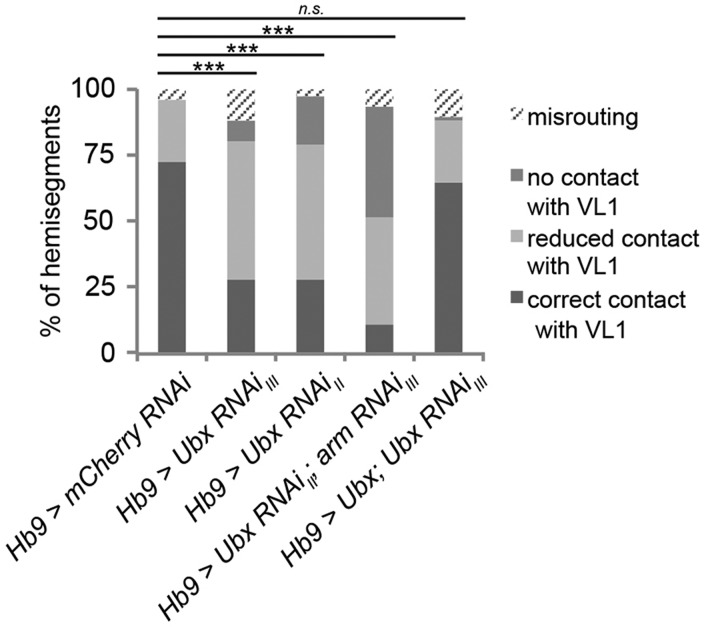
**RNAi depletion indicates cell-autonomous requirements for *Ubx* and *arm* in VL1-MNs to control correct innervation of VL1.** Shown is quantification of the VL1 innervation rate in early stage 17 embryos after knockdown of the indicated factors in MNs using *Hb9-*Gal4. Knockdown of *Ubx* leads to defects in VL1 innervation compared with a control RNAi construct (UAS-*mCherry* RNAi). The effect of the *Ubx* single knockdown can be increased by a combined knockdown of *Ubx* and *arm*. Parallel expression of a UAS-*Ubx* construct with UAS-*Ubx* RNAi leads to an almost complete rescue, indicating the specificity of the *Ubx* RNAi construct. Correct contacts were tested versus the combination of wrong contacts (misrouting, no contact with VL1, reduced contact with VL1) and significance of the data sets was tested using χ^2^-test. ***P<0.005, n.s., not significant. *Hb9-*Gal4>UAS-*mCherry* RNAi: *n*=78; *Hb9-*Gal4>UAS-*Ubx* RNAi_III_: *n*=107; *Hb9-*Gal4>UAS-*Ubx* RNAi_II_: *n*=83; *Hb9-*Gal4>UAS-*Ubx* RNAi_II_; UAS-*arm* RNAi_III_: *n*=95; *Hb9-*Gal4>UAS-*Ubx*; UAS-*Ubx* RNAi_III_: *n*=120 (n, number of evaluated hemisegments).

As *arm* is clearly required in the MNs (Fig. 5) and it has been shown previously that Ubx and Arm can interact both physically and genetically in other contexts (Bondos et al., 2006; Hsiao et al., 2014), we wondered whether *Ubx*, apart from being required in the muscles, is also required in the MNs to establish correct VL1 innervation. To this end, we performed an RNAi knockdown of *Ubx* specifically in postmitotic *Hb9*-positive MNs. Indeed, a reduction of correct contacts on VL1 to 28% (*n*=107) resembles the *Ubx* mutant phenotype and can be rescued by providing Ubx in the same cells (Fig. 7; Table S1). Moreover, a simultaneous RNAi knockdown of *Ubx* and *arm* enhances the effect of single knockdowns with only 10.5% (*n*=95) of correct contacts compared with 27.7% (UAS-*Ubx* RNAi_II_ *n*=83) and 29.6% (UAS-*arm* RNAi, *n*=142), further supporting the notion that Ubx and the Wnt4 signalling component Arm interact in VL1-MNs to ensure proper VL1 innervation. Together, our results show that *Ubx* is necessary both in muscles (to activate Wnt4 ligand expression) and in MNs (to interact with a crucial component of the signalling pathway) and therefore strongly suggest that *Ubx* coordinates Wnt4 signalling between these two cell types to establish proper neuromuscular connections.

### *Ubx* plays a dual role in muscles and neurons to ensure proper innervation of VL muscles

**Fig. 8.**
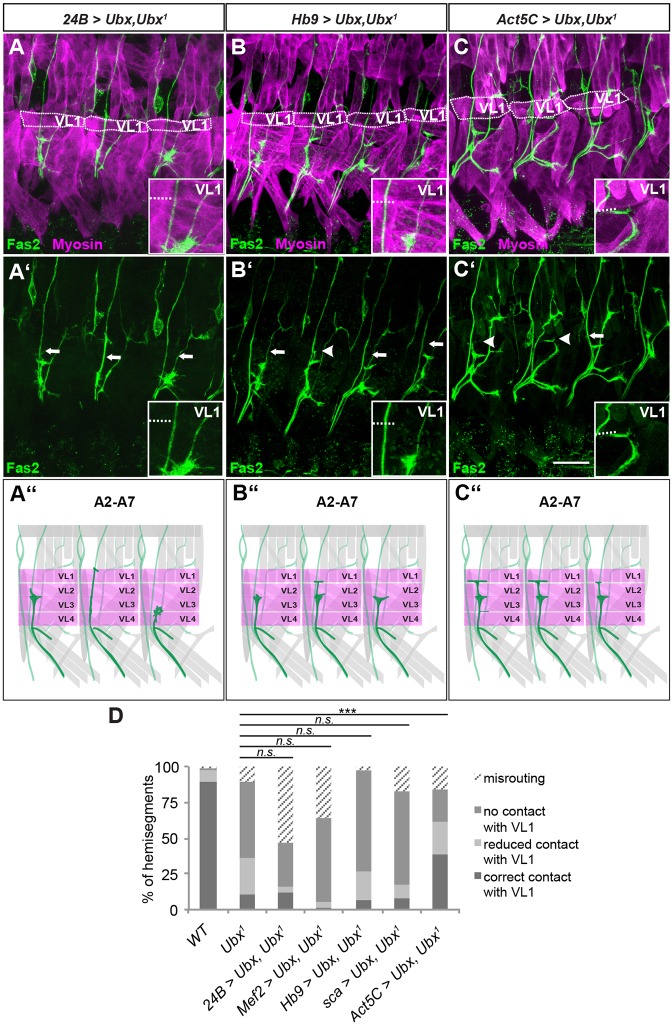
***Ubx* is needed from A2 to A7 in both VL1-MNs and VL1 muscles to ensure a correct innervation pattern.** (A-C′) Shown are filet preparations of early stage 17 embryos stained with anti-Fas2 (green) and anti-Myosin (magenta). The genotypes are given above each panel. (A,A′) Restoring expression of Ubx in all muscles using *24B*-Gal4 cannot rescue VL1 innervation defects in *Ubx* mutants (white arrows). (B,B′) Neuronal *Ubx* expression using *Hb9*-Gal4 cannot rescue VL1 innervation defects (white arrows mark defective contacts on VL1, white arrowhead marks correct contact, see inset). (C,C′) Restoring *Ubx* expression ubiquitously using *Act5C*-Gal4 rescues VL1 innervation defects (white arrow marks defective contact on VL1, see insets; white arrowheads mark correct contacts). (A″-C″) Schematics of the observed defects. ISNb is shown in dark green and the VL muscles in magenta. In A, B and C, VL1 muscles are encircled with a dotted line. In all insets, the dashed line marks the ventral VL1 border. Anterior is to the left, dorsal is up in all images. Scale bar: 20 µm. (D) Quantification of the ISNb defects in the different genetic backgrounds. Driving *Ubx* expression in an *Ubx^1^* mutant background using either muscle-specific drivers (*24B*-Gal4, *Mef2*-Gal4) or drivers for early (*sca*-Gal4) or late (*Hb9*-Gal4) neuronal expression cannot rescue VL1 defects. Only ubiquitous expression of *Ubx* in the *Ubx^1^* mutant background can rescue contacts with VL1 up to almost 40%. Correct contacts were tested versus the combination of wrong contacts (misrouting, no contact with VL1, reduced contact with VL1) and significance of the data sets was tested using χ^2^-test. ***P<0.005, n.s., not significant. WT: *n*=125; *Ubx^1^*: *n*=97; *24B*-Gal4>UAS-*Ubx*, *Ubx^1^*: *n*=75; *Mef2*-Gal4>UAS-*Ubx*, *Ubx^1^*: *n*=53; *Hb9*-Gal4>UAS-*Ubx*, *Ubx^1^*: *n*=72; *sca*-Gal4>UAS-*Ubx*, *Ubx^1^*: *n*=117; *Act5C*-Gal4>UAS-*Ubx*, *Ubx^1^*: *n*=168 (n, number of evaluated hemisegments).

To test whether *Ubx* function is indeed required in both muscles and neurons, we performed rescue experiments, restoring *Ubx* expression in a tissue-specific manner in the *Ubx* mutant background. We first tested whether expression in muscles alone would be enough to rescue the VL1 innervation phenotype. Neither of the two mesodermal drivers we used, *24B*-Gal4 and *Mef2*-Gal4, was able to restore normal VL1 innervation (Fig. 8A,D; Table S1). Expression of *Ubx* only in postmitotic MNs using the *Hb9*-Gal4 driver was also not sufficient for correct VL1 innervation (Fig. 8B,D; Table S1). To examine whether *Ubx* is needed earlier during the specification process of NB3-1, we used the *scabrous*-Gal4 (*sca*-Gal4) driver, which drives in the early neuroectoderm and remains active in most cells of the VNC until late developmental stages. However, this driver line could also not rescue aberrant VL1-MN projections in the *Ubx* mutant (Fig. 8D; Table S1).

Finally, we employed the ubiquitous *Actin5C*-Gal4 driver line (*Act5C*-Gal4) to restore *Ubx* expression in both tissues. Here, we observed a significantly higher rate of correct contacts on VL1 (38.7%, *n*=168) compared with *Ubx^1^* mutants (10.3%, *n*=97) (Fig. 8C,D; Table S1). The relatively low rescue rate is likely to be due to *Act5C*-Gal4 not being active at exactly the required developmental time points in muscles and MNs. In addition, the levels of Ubx expressed under its control, especially in the CNS, were lower than the endogenous ones (Fig. S4) and were thus unlikely to be sufficient for a more pronounced rescue of the *Ubx* phenotype. Nevertheless, partially restoring Ubx expression in both tissues shows significant rescue rates, highlighting a role for *Ubx* in coordination of the ligand trigger from muscles and the response in MNs to ensure proper innervation of VL muscles.

## DISCUSSION

In this article, we address the question of how region-specific neuromuscular connections are established along the anteroposterior body axis during development. Previous studies on *Drosophila* embryos reported segment-specific differences in the morphology of VL muscles (Bate, 1993; Hooper, 1986), thus providing a good model for our investigations. Here, we provide a more detailed characterisation of this muscle group in the thorax and anterior abdomen, including segment-specific variations in the patterns of VL innervation. VL muscles show the same morphological pattern in abdominal segments A1 to A7, but diverge from it in the thorax, with each thoracic segment exhibiting a distinct VL morphology. VL innervation follows a similar pattern distribution, with projections of the T2 and T3 VL1-MNs, which innervate VL1 muscles in T3 and A1, being alike. VL1-MNs from abdominal segments A1 to A6 innervate segments A2 to A7 and have similar projections in these segments.

### *Ubx* regulates region-specific *Wnt4* expression in VL muscles

We find these segment-specific morphological characteristics to coincide closely with the expression pattern of the Hox gene *Ubx* (Fig. 2). In VL muscles, Ubx expression is excluded from thoracic segments, is low in A1, high in A2 and declines gradually towards A7. In the RP MNs, Ubx levels are low in T2 and T3, high in A1, and are reduced gradually until A6. These patterns correlate well with previous studies that found general Hox expression boundaries to be segmental in the muscles (Bate, 1993) and parasegmental in the nervous system (Hirth et al., 1998). In *Drosophila*, Hox genes are known to act early in the mesoderm and neuroectoderm to establish region-specific patterns of muscles and neurons, respectively (Hooper, 1986; Michelson, 1994; Technau et al., 2014). Their expression continues until late embryonic stages where they play more direct roles in later developmental events in both invertebrates and vertebrates, such as neuronal survival, migration and connectivity (Philippidou and Dasen, 2013).

We show that, in segments A2 to A7, *Ubx* controls the expression of *Wnt4* in muscle VL2. Wnt4 is secreted to provide a repulsive signal to the VL1-MNs, forcing them to extend their growth cones further and synapse onto the more dorsal muscle VL1 (Inaki et al., 2007). Interestingly, VL1-MNs in segments T3 and A1 are not dependent on the Wnt4 signal. Factors such as Toll, Beat-IIIc or Glutactin have been shown to have redundant functions with Wnt4 in the abdomen (Inaki et al., 2007, 2010). We suspect that they could represent the repulsive signals in T3 and A1. *Ubx* additionally controls the expression of *Sulf1*, which has been shown to play a role in axonal targeting (Inaki et al., 2007). *Sulf1* expression is entirely dependent on *Ubx* only in segments A1 and A2. As expression in abdominal segments A3 to A7 is weaker in the *Ubx* mutant, but is not completely lost, it is reasonable to assume that *Sulf1* might be co-regulated by *Ubx* and the more posterior Hox genes *abdominal-A* and *Abdominal-B* in these segments. Expression of *Wnt4* and *Sulf1* at late stage 14 correlates well with the time point at which the growth cones enter the muscle field to find their targets (Prokop, 1999), supporting their role in axonal targeting. Evidence from several species shows that Sulf1 regulates the secretion, stability and the diffusion range of different Wnt morphogens during canonical and non-canonical Wnt signalling (Ai et al., 2003; Dhoot et al., 2001; Fellgett et al., 2015; Kleinschmit et al., 2010; Tran et al., 2012), which suggests that similar mechanisms might be at work in other organisms.

### Wnt4 signalling in neural network formation

Once secreted, Wnt4 binds and activates receptors of the frizzled family (Frizzled 2) and of the RYK family (Derailed 2) on the VL1-MNs (Inaki et al., 2007). However, further details of the mechanisms involved were not reported. Functions for Wnt signalling in neural development, including early specification of neural stem and progenitor cells (Deshpande et al., 2001; Prokop and Technau, 1994), axonal pathfinding and synapse formation (Inaki et al., 2007; Klassen and Shen, 2007; Lyuksyutova et al., 2003; Maro et al., 2009; Reynaud et al., 2015; Yoshikawa et al., 2003; Zheng et al., 2015), are well-documented in multiple species. Wnts also exhibit late effects during physiological regulation of the *Drosophila* NMJ (Kerr et al., 2014; Packard et al., 2002) and during long-term memory formation (Chen et al., 2006; Tan et al., 2013). However, activation of the canonical Wnt signalling pathway in MNs has, to our knowledge, only been described in *Caenorhabditis elegans*, in which the β-catenin homologue BAR-1 is required in a specific type of MN for Wnt-regulated axonal guidance (Maro et al., 2009). We now show a MN-specific requirement for Arm and TCF, a further downstream component of the canonical Wnt signalling pathway, in correct targeting of VL muscles.

**Fig. 9.**
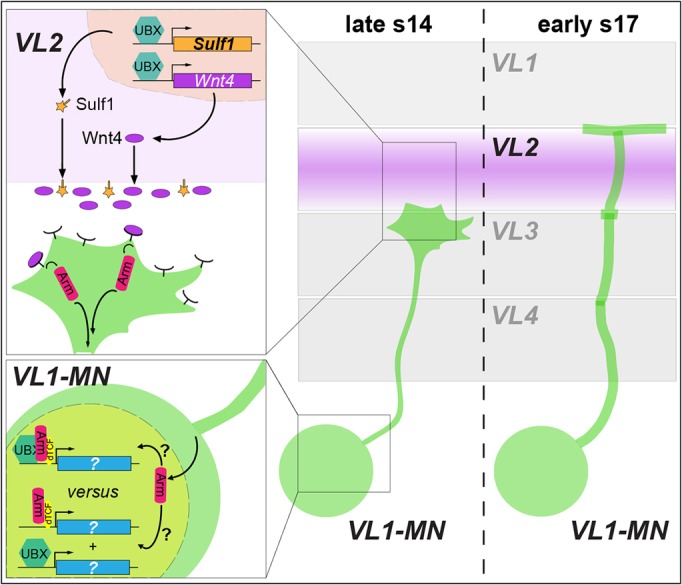
**A combined role of Ubx in muscles and neurons during muscle innervation.** During late stage 14, Ubx regulates the expression of Sulf1 and Wnt4 in muscle VL2. These factors are secreted by VL2 to signal to the arriving growth cones. In VL1-MNs, Wnt4 is sensed by its receptors Fz-2 and Drl-2, the first one probably triggering the canonical Wnt4 signalling pathway in these neurons. Arm translocates to the nucleus, where it either interacts with TCF and Ubx to control potential target genes or acts with TCF in parallel to Ubx to regulate the status of VL1-MNs. This process triggers the repulsion by the Wnt4 signal and leads to extension of VL1-MN axons towards VL1.

Furthermore, our results suggest that Ubx itself interacts with the Wnt4 signalling pathway in MNs to ensure correct targeting of muscle VL1. A recent study showed that TCF interacts specifically with Ubx but not AbdA in an *in vivo* bimolecular fluorescent complementation assay (Baëza et al., 2015). Assuming that motoneuronal targeting and synapse formation are, at least in part, transcriptionally regulated under influence of Wnt4, this difference in interaction capability might account for the different effects on VL1 innervation seen between *Ubx* and *abdA* mutants. As Ubx and Arm have been demonstrated to interact physically and genetically (Bondos et al., 2006; Hsiao et al., 2014), it is tempting to speculate that Ubx, TCF and Arm might control target genes by forming a Wnt4-induced transcriptional complex (Fig. 9). This remains to be confirmed as techniques for visualizing such complexes *in situ* are currently not available. Alternatively, the genetic interaction might be based on Ubx and Arm/TCF acting in parallel on target genes to regulate axonal targeting.

### A coordinating role for Ubx in muscle innervation

Interestingly, expressing *Ubx* either in muscles or in MNs was not able to rescue correct VL1-MN contact formation on VL1 in *Ubx* mutants. Only ubiquitous *Ubx* expression resulted in significant rescue of the phenotype, identifying *Ubx* as the key factor that coordinates production of the Wnt4 ligand in, and, through regulation of *Sulf1* expression, possibly also its secretion from, muscle VL2 with the signalling pathway response in the corresponding MNs. Both of these events are required to direct proper innervation of VL1 by VL1-MNs in abdominal segments A2 to A7. This finding corroborates a previous report on larval crawling behaviour that showed that ectopic expression of *Ubx*, when restricted only to muscles or to neurons, did not alter thoracic peristaltic movements (Dixit et al., 2008). By contrast, ubiquitous ectopic expression of *Ubx* resulted in anterior thoracic segments showing peristaltic patterns characteristic of the abdomen. These observations strongly suggested a function for *Ubx* in both tissues, and our work now provides a possible mechanism to at least partially explain them. The finding that *Ubx* coordinates expression of muscle-specific axon guidance factors on the one side, and activation of the motoneuronal response on the other, reveals a novel function for Hox genes in nervous system development. In addition, our results provide new insights into the regulation of interactions between different cell types during development, suggesting that the same transcription factor can coordinate spatially restricted generation of a signal in one type of cell, with the response to that signal in another.

Future studies will show whether this principle holds true in mammalian systems. Both Hox genes and the role of *Wnt4* in NMJ development are conserved between *Drosophila* and mammals (McGinnis and Krumlauf, 1992; Strochlic et al., 2012). Furthermore, *Wnt4* expression in mammalian muscles is temporally regulated in a manner similar to that in *Drosophila* embryos (Strochlic et al., 2012), suggesting that the same mechanism might coordinate muscle innervation in mammals.

## MATERIAL AND METHODS

### Fly stocks and genetics

The following fly stocks were used: Oregon R, *w^1118^, Ubx^1^*/TM6B, *Tb*, *Sb*, *Dfd*-lacZ and UAS-*Ubx* (kindly provided by L. S. Shashidara, IISER, Pune, India), *Ubx^6.28^*/TM6B, *Tb*, *Sb*, *Dfd*-lacZ, *abdA^MX1^*/TM3, *Sb, Kr-*Gal4, UAS*-GFP* (Sánchez-Herrero et al., 1985), *Hb9-*Gal4/TM3, *Sb*, *ftz*-lacZ (Broihier et al., 2002), *24B*-Gal4, UAS-*mCherry*/TM6B (kindly provided by S. Merabet, IGFL, Lyon, France), *Mef2*-Gal4, UAS-*CD4::td-tom.FP/*TM6B (kindly provided by O. Vef, University of Mainz, Germany), *Act5C*-Gal4/CyO, *Wnt4^EMS23^*, *bw*/CyO, *hb*-lacZ, *arm^4^*/FM7, *grh-*lacZ, *arm^8^*/FM7c*,* *Dfd*-GMR-*nvYFP*, UAS-*mCherry* RNAi, UAS-*Ubx* RNAi, UAS-*arm* RNAi, UAS-*sgg.B (*UAS*-GSK3)*, UAS-*dTCFΔN (*UAS*-dTCF.DN)*, UAS*-CD4::tdGFP* and UAS*-CD8::GFP* (all from Bloomington Stock Center, Indiana, USA). *arm^8^/X* or *arm^8^/X;; Ubx^1^/+* animals were identified by the anti-Sex lethal signal.

The UAS-*Ubx* RNAi insertion on the second chromosome (attP40) was generated using the *shUbx* RNAi (HMS01403) construct in pValium20 (Ni et al., 2011) (kindly provided by the TRiP consortium, Harvard, USA). All experiments were performed at 25°C except for the RNAi and dominant-negative experiments, which were incubated at 29°C.

### Immunohistochemistry

For antibody staining, embryos were dechorionated, fixed and immunostained following previously published protocols (Becker et al., 2016), except that embryos were fixed for 22 min. Staging of embryos was carried out according to Campos-Ortega and Hartenstein (1997). Early stage 17 was staged according to Pereanu et al. (2007).

The following primary antibodies were used: chicken anti-Beta-Gal (1:1000; Abcam, ab9361), rabbit anti-Beta-Gal (1:1000; Cappel, 55976), mouse anti-GFP (1:250; Roche, 11814460001), rabbit anti-GFP (1:500; mTorrey Pines Biolabs, TP401), mouse anti-Fas2 1D4 (1:10), mouse anti-Cut 2B10 (1:20), mouse anti-Sxl M18 (1:10), mouse anti-Ubx FP3.38 (1:20) (all from Developmental Studies Hybridoma Bank), rat anti-Myosin (1:500; Abcam, ab51098), rabbit anti-Hb9 (1:2000; kindly provided by J. B. Skeath, Washington University in St. Louis, USA) and guinea pig anti-Ubx (1:200; kindly provided by I. Lohmann, University of Heidelberg, Germany).

As fluorescent secondary antibodies we used anti-guinea pig Dylight 405, anti-chicken Alexa Fluor 647 (both Jackson ImmunoResearch), anti-mouse Alexa Fluor 488, anti-rabbit Alexa Fluor 488, anti-mouse Alexa Fluor 568, anti-rabbit Alexa Fluor 568, anti-rat Alexa Fluor 633 (all from Thermo Fisher Scientific) at 1:500. All secondary antibodies were used according to the manufacturer's protocols.

### *In situ* hybridisation

For *in situ* hybridisation, the *Sulf1* probe was generated by PCR as reported previously (Weiszmann et al., 2009). The *Wnt4* probe was made from EST clone RE26454 (Stapleton et al., 2002) upon digestion with *Bsg*I (New England Biolabs). Primer sequences are: Dsulf1-fwd, 5′-GCCTTATAATTGGCGGCC-3' and Dsulf1-rev-SP6, 5′-ATTTAGGTGACACTATAGAAGAGTTGAGGAGCGGAGGAAGG-3′. Both probes were labelled using the DIG-RNA Labelling Kit (Roche). The hybridisation on embryos was carried out as described before (Tautz and Pfeifle, 1989). Probes were detected using anti-DIG AP (Roche).

### Image acquisition

The non-fluorescent staining was documented using a Zeiss Axioplan. Fluorescent confocal images were acquired on a Leica TCS SP5 microscope. Laser intensities were kept constant between experiments and controls. Image processing was carried out using ImageJ, Adobe Photoshop CS4 and Adobe Illustrator CS4.

### Statistical analysis

Statistical analysis of categorical data was performed using χ^2^ tests for pairwise comparisons with controls. Correct contacts were tested against the combination of wrong contacts. Operators were not blind to treatment groups.
